# Perceptions of dogs and animal-assisted support programs among veterinary students and teaching staff in Romania

**DOI:** 10.3389/fvets.2026.1871706

**Published:** 2026-07-29

**Authors:** Alexandra Grigoreanu, Cristina Gaspar, Iulia Bucur, Alexandru Udrea, Razvan Cojocaru, Alex Moza, Alexandra Ban-Cucerzan, Cristian Lazarescu, Emil Tirziu, Ioan Tibru

**Affiliations:** 1 Faculty of Veterinary Medicine, University of Life Sciences “King Mihai I” from Timişoara, Timişoara, Romania; 2 Doctoral School of Veterinary Medicine, University of Life Sciences “King Mihai I” from Timisoara, Timişoara, Romania

**Keywords:** animal-assisted support programs, attitudes, dogs, higher education, mental health, survey, veterinary students

## Abstract

Animal-assisted support programs (AASP) are increasingly used to promote psychological wellbeing in educational settings, yet their acceptability within veterinary education remains underexplored. This study aimed to assess perceptions, knowledge, attitudes, and implementation preferences related to AASP among veterinary students and teaching staff from a Romanian Faculty of Veterinary Medicine. A cross-sectional survey was conducted among 200 students and 99 teaching staff members, using a structured questionnaire addressing demographics, attitudes toward dogs, awareness of AASP, and self-reported mental health-related variables. Group and gender differences were analyzed using chi-square and Fisher's exact tests. Overall, respondents demonstrated positive attitudes toward dogs and strong acceptance of AASP. Most participants perceived AASP as potentially beneficial for stress reduction, mental health support, and enhancement of the educational environment, despite limited prior experience with such programs. A considerable proportion of students (20.5%) reported a diagnosed mental health condition, highlighting the relevance of student wellbeing support within the investigated academic setting. Gender differences were observed, with female respondents generally expressing more favorable attitudes and lower risk perception. High willingness to participate and support for the potential introduction of structured AASP and related training opportunities were reported, although concerns regarding anxiety and logistical constraints were acknowledged. These findings indicate a high level of perceived acceptability of AASP among veterinary students and teaching staff and support further investigation of their potential role within student wellbeing initiatives in veterinary higher education.

## 1 Introduction

The use of dogs in therapeutic and supportive contexts has been associated with significant benefits for individuals with emotional and cognitive challenges (1). Consequently, animal-assisted support programs (AASP; formerly known as animal-assisted activities) (2) have gained increasing attention in educational settings, where they are used to improve student wellbeing, reduce stress, and enhance engagement (3).

The term Animal-Assisted Support Programs refers broadly to structured, non-clinical animal-assisted initiatives implemented within educational settings with the aim of providing social, emotional, or wellbeing-related support. This term is distinct from Animal-Assisted Therapy (AAT), which constitutes a goal-directed clinical intervention delivered by qualified health professionals, and from Animal-Assisted Activities (AAA), which generally involve less structured interactions intended to promote wellbeing, motivation, or recreational engagement (2).

In university environments, such programs have been shown to positively influence mental health by reducing stress, anxiety, and depression among students, increasing their motivation and engagement, and improving their social and performance-related skills (4–6).

However, successful implementation requires an understanding of the perspectives of key stakeholders, including both students and teaching staff (7). It is well known that teachers play an essential role in shaping the academic experience of students, and their opinions can influence the success of programs involving animals regardless of their form (8).

Despite growing interest in AASP, limited research has explored perceptions of AASP within veterinary education, a context in which familiarity with animals may influence attitudes, expectations, and feasibility considerations (9). Moreover, veterinary students represent a population at increased risk of stress, anxiety, and burnout due to the demanding nature of their training, emotional exposure to animal suffering, and academic pressure (10, 11). These factors highlight the need for accessible and preventive mental health support strategies within veterinary education (12).

This study aims to assess the perceptions, knowledge, and attitudes of veterinary students and teaching staff toward AASP, as well as their perceived benefits, risks, and feasibility within the university environment. The findings are intended to inform the development of structured, context-appropriate animal-assisted support programs in higher education.

## 2 Material and methods

The survey was conducted in the University of Life Sciences “King Mihai I” From Timisoara, Romania, with all the data collected within the target Faculty of Veterinary Medicine. The sample consisted of 200 students and 99 teaching staff members.

The questionnaire used in the present study was developed specifically for this exploratory cross-sectional research in order to assess perceptions, attitudes, awareness, and implementation preferences related to Animal-Assisted Support Programs (AASP) among veterinary students and teaching staff. The development of the questionnaire was based on a review of the available literature concerning animal-assisted interventions, veterinary student mental health, attitudes toward companion animals, and educational support strategies in higher education settings (8, 13–17).

Most questionnaire items used categorical response formats, including dichotomous (Yes/No) questions, multiple-choice items, and predefined opinion-based responses. Because the questionnaire combined multiple independent thematic domains and was primarily designed for descriptive and exploratory assessment of perceptions and attitudes related to AASP within a veterinary academic setting, rather than for measuring a single latent psychometric construct, formal validation procedures such as exploratory factor analysis and global reliability coefficients were not considered fully appropriate for the instrument as a whole.

Prior to distribution, a pilot assessment was conducted on a convenience sample of 15 individuals, including psychologists, veterinary medicine teaching staff and students, and individuals with previous experience in animal-assisted activities and interventions. Participants were asked to evaluate each item regarding clarity, comprehensibility, relevance to the objectives of the study, appropriateness for the target population, and overall questionnaire length.

Based on the feedback received during this process, 83 items were removed across the two questionnaires combined, including 41 items from the student questionnaire and 42 items from the teacher questionnaire. The refinement process particularly focused on eliminating redundant, overly specific, difficult to standardize, or insufficiently relevant items. Special emphasis was placed on removing open-ended questions, as pilot participants considered them excessively time-consuming, difficult to code consistently, and likely to generate highly heterogeneous responses that would complicate statistical analysis and reduce inter-response comparability. Several items requesting participants to explicitly describe specific mental health disorders, emotional problems, learning disabilities, or personal psychological experiences were also removed. Reviewers considered these questions potentially intrusive, ethically sensitive, and beyond the primary objectives of the study. Additionally, such items were considered likely to reduce participant comfort and completion rates while generating qualitative data that would be difficult to analyze systematically within the scope of the present research design. Questions related to detailed parenting practices, child–dog interactions, aggressive canine incidents, and individualized behavioral scenarios were similarly excluded due to their limited relevance to the central objectives of the study.

Following the refinement process, both questionnaires were reduced to a final format consisting of 45 items in the teaching staff questionnaire and 41 items in the student questionnaire. The resulting instruments were structured into three main sections. The first section included demographic and background variables such as age, gender, occupation, teaching experience, physical activity habits, and selected self-reported mental health-related information. The second section assessed respondents' attitudes toward dogs, including pet ownership, previous experiences with dogs, perceptions regarding dog behavior and welfare, and opinions related to dog presence in public or educational settings. The third section evaluated awareness, perceptions, and implementation preferences related to AASP, including perceived psychological and educational benefits, willingness to participate, organizational preferences, and perceived limitations or concerns associated with such programs. Both the preliminary and the final versions of the questionnaire are included as Supplementary material.

Before completing the questionnaires, all participants received a brief standardized explanation defining AASP as structured, voluntary, non-clinical university-based initiatives involving appropriately selected dogs and trained handlers, designed to provide social, emotional, and wellbeing-related support. This explanation was included in both the online and paper-based questionnaires to ensure that respondents interpreted AASP within the same conceptual framework before answering the related items.

The study employed a voluntary-response convenience sampling design within the targeted population. Recruitment was conducted over 3 months using several complementary approaches to increase accessibility and participation within the faculty. The online questionnaire link was first distributed through institutional e-mail and online communication channels used by students and teaching staff. Paper questionnaires were subsequently offered only to individuals who reported that they had not completed the online form, for reasons such as inability to access their institutional e-mail address, technical difficulties, or a clear preference for completing the questionnaire in paper format. This procedure was used to reduce the risk of duplicate responses and minimize response-format-related bias. Both formats contained the same questions, response options, introductory information, and informed consent statement. Paper responses were later entered into the same database as the Microsoft Forms responses, using the same coding scheme and anonymization procedures. Participation was entirely voluntary, and no incentives were provided for questionnaire completion. Completion and submission of the questionnaire were considered to represent informed consent.

Before distributing the forms in public spaces, the following inclusion criteria were applied:

- Respondents had to be aware of the purpose of the questionnaire at the time of completion.
- Respondents must not have any conflict of interest with the researchers conducting the study.
- For teachers, employment in the field of education at the time of completing the questionnaire was required.
- For students, regardless of their year of study or age, attendance at classes, seminars, or practical work was required.

The year of study was not included as a stratification variable, as the objective of the present exploratory survey was to assess overall perceptions of AASP among veterinary students rather than to compare attitudes according to curriculum stage. Moreover, academic pressure may vary not only by study year but also according to individual coping strategies, personal circumstances, and subjective perception of workload.

Based on the method of distribution, the following exclusion criteria were applied:

- Inconsistencies between answers to interdependent questions.
- More than three consecutive unanswered questions, if they were directly related to a previous question.
- Questionnaires with substantial missing demographic data preventing group allocation or statistical analysis.
- Completion of questionnaires by individuals outside the target categories for analysis.

No completed questionnaires met the exclusion criteria to a degree requiring removal from the final analysis.

To examine differences between teachers and students, as well as gender differences across groups, chi-square tests of independence were applied. When the assumption regarding minimum expected cell frequencies was not fulfilled, Fisher's exact test was used to ensure the accuracy of the statistical inference. All statistical tests were two-tailed, and statistical significance was established at *p* < 0.05. Because the study was designed primarily as an exploratory and descriptive investigation, statistical analyses were interpreted cautiously and intended mainly to identify potential associations and trends within the investigated population rather than to establish causal relationships. Given the large number of exploratory comparisons performed, no formal correction for multiple testing was applied; therefore, the possibility of Type I errors should be considered when interpreting statistically significant findings. For contingency table analyses presented in the main result tables, effect sizes were assessed using Cramer's *V* to facilitate interpretation of the magnitude of observed associations alongside statistical significance.

### 2.1 Ethical statement

This study was conducted in compliance with the institutional regulations and data protection requirements of the Ethics Committee of the Faculty of Veterinary Medicine, University of Life Sciences “King Mihai I” from Timişoara, Romania. According to the institutional policies applicable at the time of the study, anonymous and voluntary questionnaire-based surveys that did not involve clinical interventions or the collection of identifiable personal data were considered exempt from formal ethics committee approval.

Participation in the survey was entirely voluntary and anonymous. Before completing the questionnaire, all participants were informed about the objectives and purpose of the study through an introductory informed consent section included at the beginning of the Microsoft Forms questionnaire and paper-based versions. Participants were informed that completion and submission of the questionnaire implied consent for the anonymous use of the collected data for scientific research purposes.

Respondents were informed that they could decline participation or skip any question they considered sensitive or uncomfortable without any consequences. No personally identifiable information was collected during the survey process.

Questionnaires were collected and stored under confidential conditions, and access to the database was restricted exclusively to the research team. Electronic data were stored on password-protected institutional devices and used solely for scientific analysis and reporting purposes.

## 3 Results

The final sample included 99 teaching staff members and 200 students, recruited from an approximate institutional population of 110 teaching staff members and 1,100 students. The student group consisted of 129 female (64.5%), and 71 male (35.5%). Among the teaching staff, 40 were female (40.4%) and 59 were male (59.6%). The observed gender distribution was broadly consistent with demographic trends described in the European veterinary education literature and is discussed further in the Discussion section.

In terms of age distribution, most of the teachers fall into the 25–35 age group (37.4%), followed by the 46–55 (25.3%) and 36–45 age groups (19.2%), with the 56–60 and over 60 age groups being markedly underrepresented (9.1% each group). In what concerns the students, most of them are in the 22–25 age range, representing 58% of the total, followed by those aged 18–21 (35%). The remaining students, accounting for only 7%, belong to age groups over 25, reflecting a predominantly young composition, specific to the university environment.

### 3.1 Teaching staff-specific data

More than half of the teachers (51.5%) fall into the 10 years plus of work experience category, followed by the 1–5 years of experience category (31.3%), 5–10 years category (10.1%) and less than 1 year experience category (7.1%). The relationship between gender and teaching style varied across age groups, χ^2^(12) = 33.73, *p* < 0.05. Women in the 36–45 age group were more likely to adopt a student-centered style, and men were more likely to adopt a traditional style than expected. Younger men (25–35 years) preferred student-centered approaches, while women in the same age group leaned slightly toward traditional methods.

More than half of the teachers (57.6%) indicated cases of students with special needs (physical disabilities, learning difficulties, or emotional/behavioral disorders), with no significant gender-based differences, χ^2^(1) = 0.71, *p* > 0.05. However, the majority of them (83.8%) believe that this category of students represents only between 0 and 5% of the total.

When asked how often they observe mental or emotional health problems in their students, the majority of the answers were “sometimes” or “rarely,” with no gender differences, χ^2^(3) = 6.1, *p* > 0.05. About 55% of women and 49% of men reported feeling prepared to support students' mental health needs “always,” “most of the time,” or “somewhat, but with additional resources or training,” with no significant differences, χ^2^(1) = 0.32, *p* > 0.05. Perceived resource needs in supporting students' mental health also showed no significant gender differences; men emphasized professional development opportunities in order to be able to better support the students, while women highlighted collaboration with mental health services. Finally, the estimated prevalence of student mental health problems, during the academic year before completing the questionnaire, differed significantly by gender, χ^2^(4) = 13.21, *p* < 0.05. Men more often reported very low rates (< 5%), while women provided more varied estimates, including higher percentages.

### 3.2 Student-specific data

Approximately 20.5% of students reported being diagnosed with a mental health problem, while 79.5% had no such diagnosis. There was a significant association between gender and diagnosed mental health condition, χ^2^(1) = 4.58, *p* < 0.05, with women (24.8%) reporting diagnoses more frequently than men (12.7%). Although there was no significant association between diagnosed mental health issues and frequency of weekly physical activity, χ^2^(2) = 1.84, *p* > 0.05, the analysis of physical activity habits showed that 39.5% of students exercise 2–3 times a week, 24% four times or more and 36.5% of respondents exercise rarely or not at all. In terms of psychoactive medication, 15% of students take prescription drugs, while 85% do not use such treatments.

### 3.3 Results on respondents' attitudes toward dogs

This section reports general attitudes toward dogs and dog ownership, independently of respondents' perceptions of AASP. These items were included because previous experience with and attitudes toward dogs may influence perceptions of AASP.

Table 1 summarizes the number (*n*) and percentage (%) of affirmative (yes or agree) and negative responses (no or neutral or disagree) provided by teachers and students, separated by gender, across 14 survey items related to attitudes toward dogs. Chi-square statistics, *p*-values, and Cramer's *V* effect sizes are presented to indicate both the statistical significance and the magnitude of gender differences within each group and comparisons between groups.

**Table 1:** Distribution of responses to questions regarding attitudes toward dogs among students and teaching staff.

Item	Group	Yes or agree			No or neutral^a^ or disagree			Statistical analysis		
		*F*	*M*	Total	*F*	*M*	Total	χ^2^(1)	*p*-value	Cramer's *V* effect
		*n* (%)			*n* (%)					
Opinion on having pets at home	S	121 (93.8)	62 (87.32)	183 (91.5)	8 (6.2)	9 (12.68)	17 (8.5)	2.47	0.12	0.11
	T	39 (97.5)	52 (88.14)	91 (91.9)	1 (2.5)	7 (11.86)	8 (8.1)	^*^	0.14	0.17
	S vs. T							0.02	0.89	0.01
Ever owned a dog	S	119 (92.25)	59 (83.10)	178 (89)	10 (7.75)	12 (16.90)	22 (11)	3.92	0.047	0.14
	T	38 (95.0)	53 (89.83)	91 (91.9)	2 (5.0)	6 (10.17)	8 (8.1)	^*^	0.47	0.09
	S vs. T							0.63	0.43	0.05
Dogs as good companions	S	114 (88.37)	65 (91.55)	179 (89.5)	15 (11.63)	6 (8.45)	21 (10.5)	0.49	0.48	0.05
	T	37 (92.50)	53 (89.83)	90 (90.9)	3 (7.50)	6 (10.17)	9 (9.1)	^*^	0.74	0.05
	S vs. T							0.15	0.70	0.02
Currently own a dog	S	106 (82.17)	54 (76.06)	160 (80)	23 (17.83)	17 (23.94)	40 (20)	1.07	0.30	0.07
	T	30 (75.0)	48 (81.35)	78 (78.8)	10 (25.0)	11 (18.65)	21 (21.2)	0.58	0.45	0.08
	S vs. T							0.06	0.81	0.01
Should people adopt from shelters	S	118 (91.47)	60 (84.51)	178 (89)	11 (8.53)	11 (15.49)	22 (11)	2.27	0.13	0.11
	T	40 (100.0)	59 (100.0)	99 (100.0)	0	0	0	na	na	na
	S vs. T							11.75	< 0.001	0.20
Ever adopted a shelter dog	S	42 (32.56)	17 (23.94)	59 (29.5)	87 (67.44)	54 (76.06)	141 (70.5)	1.63	0.20	0.09
	T	17 (42.50)	17 (28.81)	34 (34.3)	23 (57.50)	42 (71.19)	65 (65.7)	1.98	0.16	0.14
	S vs. T							0.72	0.40	0.05
Support adopting senior dogs	S	124 (96.12)	65 (91.55)	189 (94.5)	5 (3.88)	6 (8.45)	11 (5.5)	^*^	0.20	0.10
	T	40 (100.00)	38 (64.40)	78 (78.8)	0	21 (35.60)	21 (21.2)	18.07	< 0.001	0.43
	S vs. T							17.11	< 0.001	0.24
Dogs allowed in public places	S	118 (91.47)	55 (77.46)	173 (86.5)	11 (8.53)	16 (22.54)	27 (13.5)	7.7	0.005	0.20
	T	36 (90.00)	27 (45.76)	63 (63.6)	4 (10.00)	32 (54.24)	36 (36.4)	20.16	< 0.001	0.45
	S vs. T							20.81	< 0.001	0.26
Ever bitten by a dog	S	64 (49.61)	47 (66.20)	111 (55.5)	65 (50.39)	24 (33.80)	89 (44.5)	5.1	0.02	0.16
	T	19 (47.50)	40 (67.80)	59 (59.6)	21 (52.50)	19 (32.20)	40 (40.4)	4.08	0.04	0.20
	S vs. T							0.45	0.50	0.04
Some breeds more dangerous	S	77 (59.69)	49 (69.01)	126 (63)	52 (40.31)	22 (30.99)	74 (37)	1.71	0.19	0.09
	T	26 (65.00)	53 (89.83)	79 (79.8)	14 (35.00)	6 (10.17)	20 (20.2)	9.12	0.002	0.30
	S vs. T							8.67	0.003	0.17
Small breeds easier to care for	S	48 (37.21)	32 (45.07)	80 (40)	81 (62.79)	39 (54.93)	120 (60)	1.18	0.28	0.08
	T	11 (27.50)	29 (49.15)	40 (40.4)	29 (72.50)	30 (50.85)	59 (59.6)	4.64	0.03	0.22
	S vs. T							0	1	0
Off-leash dogs in public places	S	71 (55.04)	34 (47.89)	105 (52.5)	58 (44.96)	37 (52.11)	95 (47.5)	0.94	0.33	0.07
	T	20 (50.00)	9 (15.25)	29 (29.3)	20 (50.00)	50 (84.75)	70 (70.7)	13.89	< 0.001	0.37
	S vs. T							14.42	< 0.001	0.22
Use of dogs in scientific research	S	3 (2.33)	18 (25.35)	21 (10.5)	126 (97.67)	53 (74.65)	179 (89.5)	25.84	< 0.001	0.36
	T	15 (37.50)	25 (42.37)	40 (40.4)	25 (62.50)	34 (57.63)	59 (59.6)	0.24	0.62	0.05
	S vs. T							36.47	< 0.001	0.35
Training dog for good socialization	S	120 (93.02)	64 (90.14)	184 (92)	9 (6.98)	7 (9.86)	16 (8)	0.52	0.47	0.05
	T	33 (82.50)	57 (96.61)	90 (90.9)	7 (17.50)	2 (3.39)	9 (9.1)	^*^	0.03	0.24
	S vs. T							0.1	0.75	0.02

Percentages are presented separately by gender within each group. The χ^2^ and p-values reported in the “Students” (S) and “Teaching Staff” (T) sections represent gender-based comparisons within each group, whereas the “Students vs. Teaching Staff” (S vs. T) section represents comparisons between the two occupational groups irrespective of gender.

^a^Only two questionnaire items included a neutral response option; for comparative analyses these responses were grouped with non-affirmative responses.

^*^Fischer's exact test.

Both teachers and students hold a positive attitude toward having pets at home, with 91.50% of the students and 91.90% of the teachers agreeing with pet ownership. Responses concerning previous pet dog ownership were analyzed as well, as it is known that past experiences and ownership might influence the attitude toward a specific breed or species. In the case of teachers, the distribution of affirmative responses was relatively uniform between women (95.00%) and men (89.83%). In contrast, the students' responses showed a statistically significant difference between the sexes, χ^2^(1) = 3.92, *p* < 0.05. A considerably higher number of female students (92.25%) reported having a pet dog compared to male students (83.10%).

Among teachers, almost all respondents, regardless of gender (92.5% women and 89.8% men), considered dogs to be good companions, and neutral or negative responses were marginal. Similarly, among students, there was a high consensus (89.5% in agreement), with no statistically significant differences between genders, χ^2^(1) = 0.49, *p* > 0.05. The majority of the teachers (78.8%) and students (80%) reported owning a dog at the time of completing the questionnaire, with no relevant differences between genders, χ^2^(1) = 0.58, *p* > 0.05, respectively, χ^2^(1) = 1.07, *p* > 0.05.

All the teachers (100%) were in favor of adopting dogs from shelters, whereas 11% of the students did not agree with adoption, χ^2^(1) = 11.75, *p* < 0.05. Only 29.5% of the students and 34.3% of the teachers had actually ever adopted a dog. All female teachers (100%) were in favor of adopting older dogs, while most men (64.4%) were against it, *p* < 0.001. Among students, both groups had positive opinions (94.5% overall), with no significant differences between genders.

Students were significantly more in favor than teachers of allowing dogs accompanied by their owners into public places such as shopping malls and parks, χ^2^(1) = 20.81, *p* < 0.001. Both students and teachers showed significant sex-related differences in their attitudes toward this situation. Among students, female respondents agreed more often than expected, whereas male students displayed the opposite pattern, χ^2^(1) = 7.7, *p* < 0.05. A similar but stronger association was observed among teachers, χ^2^(1) = 20.16, *p* < 0.001, with female teachers showing substantially higher agreement rates and male teachers expressing disagreement far more often than expected.

The frequency of reported negative experiences with dogs (aggressive behavior or bites) was significantly gender-associated in both groups (*p* < 0.05), with men reporting more negative experiences than women, and in similar frequencies: 67.8% of the male teachers [χ^2^(2) = 4.08] and 66.2% of the male students [χ^2^(1) = 5.1].

Overall, teachers (79.8%) were more likely to consider some breeds more dangerous than others, in comparison to the students (63%), χ^2^(1) = 8.67, *p* < 0.05, with men teachers expressing this opinion far more often than women (89.83 vs. 65.0%), χ^2^(1) = 9.1, *p* < 0.05. Among students, although men (69%) expressed this opinion more frequently than women (59.7%), the differences were not statistically significant. Male teachers were more likely to believe that small breeds are easier to care for, while women were more skeptical, χ^2^(1) = 4.64, *p* < 0.05. Among teachers, women were significantly more in favor of the idea of allowing off-leash walking in public spaces such as beaches and parks than men, χ^2^(1) = 13.89, *p* < 0.001. Among students, women (55%) supported off-leash dog walking more often than men (48%), but the differences were not statistically significant.

Students were significantly less in favor of using dogs in scientific research than teachers, χ^2^(1) = 36.47, *p* < 0.001, with over 97% of women and almost 75% of male students disagreeing, χ^2^(1) = 25.84, *p* < 0.001. Teachers agreed in high proportions with the idea of training dogs for socialization, but men gave a significantly higher number of affirmative answers, *p* < 0.05. Among students, the majority were in favor of training (92% overall), with no statistically significant difference between the sexes.

### 3.4 Results of knowledge related to Animal-Assisted Support Programs (AASP)

Table 2 presents the distribution of responses from students and teachers regarding their awareness, perceptions, and attitudes toward Animal-Assisted Support Programs (AASP), as well as perceived educational benefits and practical considerations related to their implementation. Chi-square statistics, *p*-values, and Cramer's *V* effect sizes are presented to indicate both the statistical significance and the magnitude of gender differences within each group and comparisons between groups.

**Table 2:** Distribution of responses to questions regarding knowledge, perceptions, and implementation preferences related to Animal-Assisted Support Programs (AASP) among students and teaching staff.

Item	Group	Yes or agree			No or disagree			Statistical analysis		
		*F*	*M*	Total	*F*	*M*	Total	χ^2^(1)	*p*-value	Cramer's *V* effect
		*n* (%)			*n* (%)					
General awareness and perception on AASP										
Heard of AASP before	S	123 (95.35)	8 (11.27)	131 (65.5)	6 (4.65)	63 (88.73)	69 (34.5)	143.2	< 0.001	0.85
	T	40 (100.0)	59 (100.0)	99 (100.0)	0	0	0	na	na	na
	S vs. T							44.4	< 0.001	0.39
Ever participated in AASP	S	17 (13.18)	8 (11.27)	25 (12.5)	112 (86.82)	63 (88.73)	175 (87.5)	0.15	0.70	0.03
	T	4 (10.0)	7 (11.86)	11 (11.11)	36 (90.0)	52 (88.14)	88 (88.89)	^*^	1	0.03
	S vs. T							0.12	0.73	0.02
Are AASP generally beneficial	S	127 (98.45)	63 (88.73)	190 (95.0)	2 (1.55)	8 (11.27)	10 (5.0)	^*^	0.004	0.21
	T	40 (100.0)	59 (100.0)	99 (100.0)	0	0	0	na	na	na
	S vs. T							^*^	0.03	0.13
AASP as a treatment option	S	90 (69.77)	38 (53.52)	128 (64.0)	39 (30.23)	33 (46.48)	72 (36.0)	5.25	0.02	0.16
	T	39 (97.5)	46 (77.97)	85 (85.86)	1 (2.5)	13 (22.03)	14 (14.14)	7.49	0.006	0.28
	S vs. T							15.44	< 0.001	0.23
Can pets reduce stress	S	125 (96.9)	63 (88.73)	188 (94.0)	4 (3.1)	8 (11.27)	12 (6.0)	^*^	0.03	0.16
	T	39 (97.5)	58 (98.3)	97 (97.98)	1 (2.5)	1 (1.7)	2 (2.02)	^*^	1	0.03
	S vs. T							^*^	0.15	0.09
Perceived suitability of AASP in school settings										
Are AASP suitable for schools	S	122 (94.57)	66 (92.96)	188 (94.0)	7 (5.43)	5 (7.04)	12 (6.0)	^*^	0.76	0.03
	T	39 (97.5)	52 (88.14)	91 (91.92)	1 (2.5)	7 (11.86)	8 (8.08)	^*^	0.14	0.17
	S vs. T							0.46	0.50	0.04
Distract students	S	31 (24.03)	15 (21.13)	46 (23.0)	98 (75.97)	56 (78.87)	154 (77.0)	0.22	0.64	0.03
	T	11 (27.5)	22 (37.29)	33 (33.33)	29 (72.5)	37 (62.71)	66 (66.67)	1.03	0.31	0.10
	S vs. T							3.64	0.056	0.11
Perceived suitability of AASP in higher education										
Interested in participating in AASP	S	123 (95.35)	62 (87.32)	185 (92.5)	6 (4.65)	9 (12.68)	15 (7.5)	4.25	0.04	0.15
	T	37 (92.5)	50 (84.75)	87 (87.88)	3 (7.5)	9 (15.25)	12 (12.12)	^*^	0.35	0.12
	S vs. T							1.72	0.19	0.08
Students interested in participating	T	34 (85.0)	50 (84.75)	84 (84.85)	6 (15.0)	9 (12.25)	15 (15.15)	0	1	0
Teachers benefit from AASP?	S	125 (96.9)	64 (90.14)	189 (94.5)	4 (3.1)	7 (9.86)	11 (5.5)	^*^	0.06	0.14
Educational and psychological outcomes associated with AASP										
Positive educational environment	S	125 (96.9)	66 (92.96)	191 (95.5)	4 (3.1)	5 (7.04)	9 (4.5)	^*^	0.28	0.09
	T	38 (95.0)	50 (84.75)	88 (88.89)	2 (5.0)	9 (12.25)	11 (11.11)	^*^	0.19	0.16
	S vs. T							4.64	0.03	0.12
Help with academic pressure	S	120 (93.02)	62 (87.32)	182 (91.0)	9 (6.98)	9 (12.68)	18 (9.0)	1.82	0.18	0.10
	T	33 (82.5)	50 (84.75)	83 (83.84)	7 (17.5)	9 (15.25)	16 (16.16)	0.09	0.76	0.03
	S vs. T							3.37	0.07	0.11
Help with learning difficulties	S	94 (72.87)	45 (63.38)	139 (69.5)	35 (27.13)	26 (36.62)	61 (30.5)	1.94	0.16	0.10
	T	34 (85.0)	42 (71.19)	76 (76.77)	6 (15.0)	17 (28.81)	23 (23.23)	2.55	0.11	0.16
	S vs. T							1.73	0.19	0.08
Improve students' mental health	S	120 (93.02)	63 (88.73)	183 (91.5)	9 (6.98)	8 (11.27)	17 (8.5)	1.08	0.30	0.07
	T	39 (97.5)	56 (94.92)	95 (95.96)	1 (2.5)	3 (5.08)	4 (4.04)	^*^	0.65	0.06
	S vs. T							2.02	0.16	0.08
Reduce dropout rates	S	100 (77.52)	52 (73.24)	152 (76.0)	29 (22.48)	19 (26.76)	48 (24.0)	0.46	0.50	0.05
	T	35 (87.5)	41 (69.49)	76 (76.77)	5 (12.5)	18 (30.51)	23 (23.23)	4.33	0.04	0.21
	S vs. T							0.02	0.89	0.01
Practical considerations and potential concerns										
Are AASP cost-effective	S	122 (94.57)	65 (91.55)	187 (93.5)	7 (5.43)	6 (8.45)	13 (6.5)	^*^	0.55	0.06
	T	36 (90.0)	53 (89.83)	89 (89.9)	4 (10.0)	6 (10.17)	10 (10.1)	^*^	1	0
	S vs. T							1.21	0.27	0.06
Need for training	S	126 (97.67)	63 (88.73)	189 (94.5)	3 (2.33)	8 (11.27)	11 (5.5)	^*^	0.01	0.19
	T	37 (92.5)	50 (84.75)	87 (87.88)	3 (7.5)	9 (15.25)	11 (12.12)	^*^	0.35	0.12
	S vs. T							3.15	0.08	0.10
Permanent or different animals		Permanent animal			Different animals					
	S	80 (62.02)	37 (52.11)	117 (58.5)	49 (37.98)	34 (47.89)	83 (41.5)	1.85	0.17	0.10
	T	30 (75.0)	36 (61.02)	66 (66.67)	10 (25.0)	23 (38.98)	33 (33.33)	2.1	0.15	0.15
	S vs. T							1.86	0.17	0.08
Anxiety around animals	S	103 (79.84)	50 (70.42)	153 (76.5)	26 (20.16)	21 (29.58)	47 (23.5)	2.26	0.13	0.11
	T	29 (72.5)	41 (69.49)	70 (70.71)	11 (27.5)	18 (30.51)	29 (29.29)	0.1	0.75	0.03
	S vs. T							1.17	0.28	0.06
Mandatory AASP sessions	S	34 (26.36)	18 (25.35)	52 (26)	95 (73.64)	53 (74.65)	148 (74)	0.02	0.89	0.01
	T	11 (27.5)	16 (27.12)	27 (27.27)	29 (72.5)	43 (72.88)	72 (72.73)	0	1	0
	S vs. T							0.06	0.81	0.01

Percentages are presented separately by gender within each group. The χ^2^ and p-values reported in the “Students” (S) and “Teaching Staff” (T) sections represent gender-based comparisons within each group, whereas the “Students vs. Teaching Staff” (S vs. T) section represents comparisons between the two occupational groups irrespective of gender.

^*^Fischer's exact test.

Overall, respondents demonstrated a high level of awareness and positive perceptions regarding Animal-Assisted Support Programs (AASP). All teachers reported having heard of AASP prior to completing the questionnaire, whereas approximately two-thirds of students reported prior awareness (65.5%). This difference between students and teachers was statistically significant χ^2^(1) = 44.4, *p* < 0.001. Despite differences in awareness, prior direct participation in AASP was relatively limited in both groups, with only about one in eight students (12.5%) and a similar proportion of teachers (11.1%) reporting previous experience; no significant group differences were observed.

Perceptions regarding the benefits of AASP were overwhelmingly positive. Nearly all students (95.0%) and all teachers (100%) considered AASP to be generally beneficial, although a small but significant gender difference was observed among students, with male students expressing slightly more skepticism (*p* < 0.05). Similarly, a majority of respondents indicated that they would consider AASP as a treatment option, although teachers expressed significantly stronger support (85.9%) compared to students (64.0%), χ^2^(1) = 15.44, *p* < 0.001, with females being significantly more in favor than males among both groups (*p* < 0.05). Most respondents also believed that pets can help reduce stress levels, with agreement reported by 94.0% of students and 98.0% of teachers.

Approximately 94.0% of students and 91.9% of teachers agreed that AASP are suitable for schools, with no significant differences between the two groups. Only 23.0% of students and 33.3% of teachers expressed concerns about students being distracted from their studies, indicating that respondents generally perceived AASP as compatible with academic activities.

When asked which age group might benefit most from animal-assisted therapy, responses differed significantly between teachers and students χ^2^(4) = 30.58, *p* < 0.001. The teachers selected “I don't know” significantly more often than expected (*R* = 3.68), whereas students selected this option significantly less often than expected (*R* = −2.59). Teachers were also less likely than expected to identify higher education as the primary beneficiary group (*R* = −2.10). A second analysis examining gender differences also revealed a significant association χ^2^(4) = 14.72, *p* < 0.05; men selected “I don't know” more frequently than expected (*R* = 2.22), while women selected this option less often than expected.

Interest in participating in AASP within the university context was high among both groups. A large majority of students (92.5%) and teachers (87.9%) reported that they would be interested in participating in such programs. Moreover, female students expressed their interest in a significantly higher proportion than the male students (*p* < 0.05). Additionally, most teachers believed that students would be interested in participating in AASP (84.9%). From the students' perspective, 94.5% believed that teachers could also benefit from implementing AASP within the university environment.

Respondents generally perceived AASP as having positive educational and psychological effects. The vast majority of participants agreed that AASP could help create a more positive educational environment, with 95.5% of students and 88.9% of teachers endorsing this view. Students were slightly more likely than teachers to report this perception, and this difference reached statistical significance χ^2^(1) = 4.64, *p* < 0.05.

Similarly, most respondents believed that AASP could help students cope better with academic pressure (91.0% of students and 83.8% of teachers) and improve students' mental health (91.5% of students and 96.0% of teachers), although these differences were not statistically significant. A majority of respondents also believed that AASP could support students with learning difficulties (69.5% of students and 76.8% of teachers). Furthermore, around three-quarters of participants considered that AASP might contribute to reducing university dropout rates (76.0% of students and 76.8% of teachers), although a gender difference among teachers (87.5% of the females and 69.5% of the males) was observed for this item (*p* < 0.05).

Most respondents perceived AASP as a cost-effective approach for improving participants' mental health. Agreement with this statement was reported by 93.5% of students and 89.9% of teachers, with no significant differences between groups. Additionally, the vast majority of respondents believed that a specific training course would be necessary for those participating in AASP sessions (94.5% of students and 87.9% of teachers). A significant gender difference was observed among students (*p* < 0.05) for this item, with females supporting the need for training in a higher proportion than men.

Preferences regarding the organization of AASP indicated moderate variation. When asked whether institutions should have permanent support animals or bring in different animals for each session, most respondents favored permanent animals (58.5% of students and 66.7% of teachers), although these differences were not statistically significant. A substantial proportion of participants also acknowledged that some students might feel anxious around animals (76.5% of students and 70.7% of teachers), indicating awareness of potential barriers to implementation.

Regarding whether animal-assisted activities should be mandatory for all students, most respondents opposed this idea. Only about one-quarter of both students (26.0%) and teachers (27.3%) supported mandatory participation, suggesting a general preference for voluntary engagement.

Respondents' opinions regarding the preferred frequency of AASP sessions differed significantly between teachers and students χ^2^(4) = 18.61, *p* < 0.001. Teachers were more likely than expected to select less frequent session options, such as “several times per semester” (*R* = 2.92) and “once per semester or year” (*R* = 2.01), and were also more likely to report having no opinion (*R* = 2.01). In contrast, students were less likely than expected to select “several times per semester” (*R* = −2.05), indicating differing expectations regarding program frequency. The gender analysis in preferred session frequency revealed no statistically significant differences χ^2^(4) = 9.01, *p* > 0.05.

## 4 Discussion

### 4.1 Relevance to the study objectives and general considerations

Our study aimed to investigate the perspectives of veterinary students and teaching staff from a Romanian veterinary faculty regarding Animal-Assisted Support Programs (AASP) and their perceived educational and psychological relevance within an academic context. Given the exploratory nature of the study and the single-institution voluntary-response sampling design, the findings should primarily be interpreted as preliminary data reflecting perceptions within the investigated academic environment rather than as fully generalizable conclusions applicable to all veterinary higher education settings. Nevertheless, the results provide useful contextual insights regarding attitudes toward AASP, perceived mental health needs, and potential implementation considerations within veterinary education. Because participation was voluntary and based on convenience sampling, the proportion of teaching staff respondents was substantially higher relative to their representation within the institutional population structure. Consequently, teaching staff perspectives may be proportionally overrepresented in comparisons between groups, and these findings should therefore be interpreted cautiously.

The strong feminization of veterinary medicine faculties in Europe is well documented, with reports of 65%−90% female students depending on country and year (18–20). In contrast, female representation among teaching staff remains lower than that of the predominantly female student population. This pattern aligns with observations in veterinary and broader higher education systems across Europe, where women hold approximately 45%−50% of academic positions overall, although their representation decreases at senior academic levels (21). Although the observed gender distribution in the present study was broadly consistent with these demographic trends, this observation should be interpreted only as contextual support for the plausibility of the sample structure and not as evidence of full representativeness of the target population. Detailed institutional demographic reference data beyond approximate population size were limited at the time of analysis.

The age distribution also reflected expected patterns within the university context. Most students were aged 22–25 years, while teaching staff were concentrated in early and mid-career age categories. Older academic staff were underrepresented, which may influence findings related to teaching style and perceptions of student wellbeing.

The relationship between teaching style, age, and gender suggested that pedagogical approaches may differ across demographic groups. Women aged 36–45 in our sample were more likely to adopt student-centered teaching styles, whereas men were more likely to favor traditional teaching approaches. Interestingly, younger male teachers showed a greater preference for student-centered approaches than older male colleagues, suggesting a possible generational shift in educational philosophy. These findings are consistent with previous research in medical education, where female academics have been shown to adopt more student-focused and interactive teaching methods, such as small-group discussions, case-based learning, and mentoring, while male counterparts more often rely on traditional, didactic lecture-based formats (22). In veterinary education, these gender-based differences may also reflect structural inequalities in academic career progression. Although the veterinary profession has undergone substantial feminization, with women representing approximately 80% of students in many countries, men remain disproportionately represented in senior, tenured, and leadership positions within academia (21, 23). Such senior roles are often associated with more traditional, hierarchical teaching structures, whereas women are more frequently employed in clinical or laboratory-based and lower-ranked academic positions that involve direct student interaction and may encourage more student-centered teaching practices (23). At the same time, veterinary medical education has undergone a major pedagogical transformation over the past 50 years, shifting from a rigid, lecture-based model toward a more flexible, student-centered, and competency-based approach that incorporates active learning, early clinical exposure, and technological innovation (24). The stronger preference for student-centered methods among younger male teachers may therefore reflect the influence of these broader curricular and cultural changes in veterinary education. Taken together, these gender and age-related differences in teaching style may influence openness to interventions such as AASP, which are inherently student-centered and wellbeing-oriented. Teaching staff who already value interactive, supportive, and student-focused pedagogies may be more likely to perceive such interventions positively and adopt them in practice.

### 4.2 Teaching staff-specific data

Teaching staff demonstrated awareness of student vulnerabilities, with more than half reporting the presence of students with special educational or psychological needs. However, most teachers estimated that these students represented only a small proportion of the student population and reported observing mental health problems only “sometimes” or “rarely.” Moreover, only around half of the respondents reported feeling prepared to support students with mental health problems. Our results suggest that although teaching staff are often in a position to identify distressed students, they may lack sufficient training, confidence, or institutional support to intervene effectively. Visible or disclosed cases may be easily recognized, whereas less apparent psychological difficulties, such as anxiety and depression, are often internalized, leading to an underestimation of students who are “suffering in silence” (10, 25). The observation that teachers only notice issues occasionally suggests that they may not be trained to spot early warning signs, or that students, fearing stigma or academic repercussions, do not disclose their struggles, notes research into pluralistic ignorance in veterinary settings (11, 26). Besides staff training and institutional mental health resources, AASP have been proposed as one of several approaches that may contribute to creating supportive educational environments and encouraging student engagement with wellbeing initiatives (9, 12, 27–29). Given the high levels of perceived acceptance observed in the present study, future research may further explore the potential role of such programs within veterinary education settings.

In our study, gender differences in perception were also evident. Female teachers provided more varied and often higher estimates of student mental health problems, while male teachers more frequently reported very low prevalence estimates. Additionally, women emphasized collaboration with mental health services, whereas men highlighted professional development opportunities, suggesting differing support priorities. The gender differences observed among veterinary teaching staff may be related to broader gender-related differences in mental health literacy, perceived social support, and help-seeking attitudes described in recent literature (30–32). Studies suggest that women generally report greater awareness of mental health problems, higher emotional sensitivity, and stronger recognition of psychological distress, which could partially contribute to the pattern observed among female teachers (30, 33). Women are also more likely to value interpersonal and collaborative forms of support, consistent with their emphasis on collaboration with mental health services in this study (34). In contrast, men may be more likely to underestimate or under recognize psychological difficulties due to lower mental health literacy, social stigma, or gender norms that discourage acknowledgment of emotional vulnerability (31). Research indicates that men often show less favorable attitudes toward psychological help-seeking and may prioritize self-reliance or practical skill-building over external support systems (34). These factors may partly contextualize the gender-related differences observed in perceived student mental health needs.

### 4.3 Student-specific data

Approximately one in five students (20.5%) in our study reported having been diagnosed with a mental health condition, with female students reporting it significantly more frequently than males, and 15% of the students reported using psychoactive medication. Although no significant association was found between diagnosed mental health conditions and frequency of physical activity, more than one-third of students reported exercising rarely or not at all.

The self-reported mental health indicators observed in the present study are consistent with previous literature describing veterinary students as a high-risk population for stress, anxiety, depression, and burnout due to academic pressure, emotional demands, and professional uncertainty, compared to the general population and students in other disciplines (35–37). Humer et al. (35) reported a high prevalence of clinically relevant mental health symptoms among Austrian veterinary medicine students, including symptoms of depression, anxiety, insomnia, and stress, with women generally reporting higher symptom severity than men. Similarly, Wells et al. (37) found strong associations among stress, anxiety, and depression in veterinary medicine students, suggesting these mental health issues often co-occur rather than exist independently. Daughaday-Provine (38) and Neubauer et al. (39) further demonstrated elevated levels of stress, anxiety, and burnout among undergraduate veterinary students, indicating that psychological burden begins early and extends beyond veterinary medicine programs to related educational tracks.

Veterinary education is characterized by unique academic and emotional stressors, including heavy workload, performance pressure, financial concerns, and uncertainty regarding future careers (10, 39, 40). The higher proportion of self-reported mental health conditions among female students in the present study is consistent with findings reported in previous veterinary education research indicating greater psychological distress and potentially higher suicide-related vulnerability among women in veterinary education (35, 36). Protective factors such as emotional intelligence, adaptive coping strategies, and institutional support may mitigate these effects (10, 37, 40). In this context, the strong support for Animal-Assisted Support Programs observed in the present study may reflect the need for innovative, accessible, and preventive mental health interventions in veterinary education.

### 4.4 Respondents' attitudes toward dogs

In our study, overall attitudes toward dogs were strongly positive among both teachers and students, with limited gender differences in general perceptions, ownership, adoption attitudes, and companionship value. Most respondents supported pet ownership, considered dogs good companions, and currently owned a dog. These findings are consistent with previous studies reporting generally positive attitudes toward companion animals among veterinary students and professionals (13, 41–43). Such attitudes are expected within veterinary education, where students and teaching staff are frequently exposed to animals both academically and personally. As noticed by Kogan et al. (13), despite positive attitudes toward companion animals, veterinary students face practical challenges in dog ownership due to demanding schedules, with 76% expressing desire for on-campus dog care facilities to support their wellbeing.

Analysis of previous pet ownership among teachers revealed evenly distributed responses between men and women, with women slightly more likely to report previous ownership. In contrast, students showed a significant gender difference, with more female students reporting ownership of a pet dog than male students. Dijkstra Klaasse et al. (44) saw these patterns as well, that female veterinary students expressed significantly higher attitudes toward pets compared to their male peers, albeit with a small effect size.

Support for shelter adoption was also high, particularly among teachers (100% agreement). However, actual adoption rates (29.5% of the students and 34.3% of the teachers) were considerably lower than the expressed support, suggesting a discrepancy between attitudes and behavior that may reflect practical or lifestyle constraints. While supporting the concept, veterinary students and teachers often have busy schedules, unpredictable working hours, and potentially limited living space, making pet ownership difficult (10, 13, 45). On the other hand, professionals are acutely aware of the prevalence of behavioral issues (separation anxiety, aggression) and medical problems (parasites, infectious diseases like kennel cough) in shelter animals, which may cause hesitation compared to obtaining a pet with a known history (46).

Several gender-related differences emerged. Female respondents generally expressed more positive and welfare-oriented attitudes, including stronger support for adoption of older dogs, allowing dogs in public places, and off-leash walking. Male respondents reported more negative experiences with dogs and were more likely to perceive certain breeds as dangerous or to support the use of dogs in scientific research.

Our finding that all female teachers supported adopting older dogs, while one third of male teachers were opposed, aligns with previous research showing that women, in general, demonstrate greater willingness to adopt dogs compared to men (47). Mueller (48) observed that older men who care for aging companion dogs develop greater empathy toward the aging process, suggesting that attitudes toward adopting older dogs may shift with life stage. This could explain why gender differences were strongly expressed among teachers but not among younger students.

The finding that female teachers were significantly more supportive of allowing off-leash dog walking compared to male teachers is consistent with prior research showing that women place greater value on off-leash dog areas in urban green spaces (49). Among students, the gender difference was smaller and not statistically significant, aligning with Šedivá et al. (50), who found no substantial gender effect on the frequency of unleashed dogs in public spaces.

Men in both groups reported significantly more negative experiences with dogs, such as aggression or bites. Women have been found to engage in more affiliative and communicative interactions with dogs (51), which may help reduce negative encounters. They are also less likely to develop fear responses following threatening incidents (52). These previous findings may help contextualize the higher frequency of negative dog-related experiences reported by male respondents.

### 4.5 Knowledge related to AASP

One of the most notable findings of this study is the high level of awareness and acceptance of AASP. All teachers and approximately two-thirds of students had heard of AASP prior to the survey, and nearly all respondents considered such programs beneficial. Despite this high awareness, direct prior participation was limited in both groups, suggesting that access to such programs remains low. Therefore, the positive perceptions observed may represent unmet demand rather than familiarity through direct experience.

The present findings are consistent with observations from veterinary and higher education research indicating that students and educators often express positive attitudes toward emerging educational or wellbeing-related initiatives despite having limited direct experience with them (14, 53–57). The combination of high awareness, strong acceptance, and low prior participation observed in the present study may therefore reflect interest in such programs and limited opportunities for direct exposure within the current educational environment. Comparable findings have been reported across veterinary, health, and educational disciplines, where respondents expressed strong interest in topics such as telehealth, ADHD, or autism-related training despite limited training opportunities (56, 58, 59). Previous research has attributed such gaps between interest and implementation to factors including curricular constraints, resource limitations, and the relatively novel nature of these initiatives within educational settings (14, 60, 61). Collectively, these studies support the importance of increasing opportunities for engagement with specialized and interdisciplinary topics while acknowledging that positive perceptions do not necessarily reflect practical experience or implementation readiness (14, 53, 55, 56).

Teachers were significantly more likely than students to consider AASP as a treatment option. This difference may be related to greater familiarity with structured therapeutic interventions, although familiarity was not directly assessed in the present study. Female respondents in both groups were generally more supportive than males, consistent with the broader pattern of greater female support for mental health-related initiatives (30, 33, 34).

Respondents perceived AASP as beneficial not only for mental health but also for educational outcomes. The majority of teachers (85%) believed students would be very interested in participating, while almost 95% of students believed teachers would also benefit from AASP. Most respondents perceived AASP as potentially helpful for stress reduction and supporting coping with academic pressure, creating a more positive educational environment, and supporting students with learning difficulties. Additionally, approximately three-quarters of respondents believed that AASP could potentially support students at risk of disengagement. These findings suggest that respondents perceived AASP as potentially supportive, wellbeing-oriented approaches that may potentially support academic retention and student engagement (61–63). Given the high stress environment of veterinary education, these perceptions may support consideration of pilot AASP initiatives in university settings.

Although both groups expressed strong support for AASP, students generally showed greater enthusiasm regarding participation frequency and perceived educational benefits. Teachers were more cautious and more likely to select less frequent session options or indicate uncertainty regarding the most appropriate beneficiary groups. Most respondents opposed mandatory participation in AASP, indicating a preference for voluntary engagement and favored the use of permanent support animals rather than introducing different animals at each session, suggesting a preference for continuity and familiarity. This is particularly relevant given that many respondents acknowledged that some students may feel anxious around animals. In a study conducted by Verhoeven et al. (8), aiming to gain insight into teachers' and parents'/caregivers' perceptions of the impacts of a specific form of Animal-Assisted Service program for primary school students, preferences for weekly or multiple monthly sessions and the desire for a permanent animal mirror practical considerations raised in qualitative work, where sustained interaction and predictable animal presence are perceived as essential for therapeutic impact. In a study made by Baird et al. (64) it has been reported that school staff, teachers and administrators express recurring worries about student safety, allergies, fear or anxiety around animals. These include concerns about whether some students might be anxious around animals, or whether allergens could trigger medical issues.

### 4.6 Study limitations

Despite the findings suggesting positive perceptions and substantial acceptance of AASP within the investigated veterinary academic environment, several limitations should be considered when interpreting the results. First, the study employed a single-institution voluntary-response convenience sampling design, which limits the generalizability of the findings to other universities or educational contexts. The veterinary academic environment may also represent a particularly favorable context for AASP, as students and teaching staff are generally familiar with companion animals and may already have positive attitudes toward dogs. Therefore, the high acceptance observed in the present study may partly reflect the existing culture of veterinary education rather than perceptions of structured support programs alone. This context should be considered when extrapolating the findings to non-veterinary academic settings.

Second, teaching staff were proportionally overrepresented relative to students compared with the institutional population structure, representing almost the entire institutional teaching population; consequently, comparisons between these groups should be interpreted cautiously, as some observed differences may partly reflect differences in sampling structure rather than true differences in attitudes or perceptions. In addition, information regarding students' year of study was not collected. Consequently, it was not possible to assess whether the participating students were representative of the overall student population across different stages of the veterinary curriculum, nor to evaluate the potential influence of curriculum stage on the observed perception.

Third, the cross-sectional design prevents causal inference, and because participation was voluntary, the possibility of self-selection bias cannot be excluded, particularly among respondents with a stronger interest in mental health topics, companion animals, or animal-assisted interventions.

Moreover, a relatively large number of exploratory statistical comparisons were performed without formal correction for multiple testing, which may increase the possibility of Type I errors. Consequently, statistically significant associations should be interpreted cautiously and primarily as exploratory findings requiring further confirmation in larger and more analytically robust studies.

Additionally, most participants had limited direct experience with AASP, meaning responses may reflect expectations rather than informed evaluations.

Furthermore, certain demographic groups, particularly older teaching staff, were underrepresented, which may have influenced findings related to teaching styles, perceptions of student wellbeing, and openness to innovative interventions such as AASP. Finally, the reported prevalence of mental health conditions and psychoactive medication use should be interpreted as self-reported indicators rather than clinical prevalence estimates, as no standardized diagnostic or screening instruments were employed.

## 5 Conclusions

The present study found that a considerable proportion of participating veterinary students reported previously diagnosed mental health conditions, while both students and teaching staff show strong acceptance of Animal-Assisted Support Programs (AASP) as a potential supportive and wellbeing-oriented initiative within the educational environment. Positive attitudes toward dogs, high perceived benefits of AASP, and strong willingness to participate indicate positive perceptions regarding the potential acceptability and perceived value of such interventions within veterinary higher education. Structured, voluntary, and well-designed AASP warrant further investigation as a potential complementary component of mental health and student support initiatives in veterinary higher education. These findings primarily provide evidence of the acceptability and perceived value of AASP among respondents and support the need for future feasibility studies and pilot implementations within veterinary higher education settings.

## Data Availability

The original contributions presented in the study are included in the article/Supplementary material, further inquiries can be directed to the corresponding authors.
