# Supplementary material for: Perceptions of dogs and animal-assisted support programs among veterinary students and teaching staff in Romania
Source: Front Vet Sci. 2026 Jul 29;13:1871706. doi: 10.3389/fvets.2026.1871706 (PMC13461325; doi:10.3389/fvets.2026.1871706)
Supplement: Supplementary file 1 [file Data_Sheet_1.pdf]

Student Questionnaire

1. What is your age?
   a. 18–21
   b. 22–25
   c. 26–30
   d. 31–35
   e. 36+
2. What is your gender?
   a. Female
   b. Male
3. Are you currently enrolled in school?
   a. Yes
   b. No, but I plan to return to school
   c. No, and I do not plan to return to school
4. What is your current level of education?
   a. High school diploma (without baccalaureate)
   b. High school diploma (with baccalaureate)
   c. Bachelor's degree
   d. Master's degree
   e. Doctoral degree
5. Are you currently employed?
   a. Yes
   b. No
   c. Prefer not to say
6. Are you currently married?
   a. Yes
   b. No
   c. Prefer not to say
7. Do you have children?
   a. Yes, one child
   b. Yes, two children
   c. Yes, three or more children
   d. No
8. How often do you exercise per week?
   a. Never
   b. Once a week
   c. 2–3 times a week
   d. 4–5 times a week
   e. More than 5 times a week
9. Have you ever been diagnosed with a health problem (mental or emotional)?
   a. Yes
   b. No
10. If yes, what diagnosis do you have?
    ………………………………………….
11. Are you currently taking prescription medication?
    a. Yes
    b. No
12. What hobbies or activities do you enjoy?
    ………………………………………….
13. Do you have children?
    a. Yes
    b. No
14. Have you talked with your children about safe interaction with dogs?
    a. Yes
    b. No
15. Has your child ever expressed fear or anxiety related to dogs?
    a. Yes
    b. No
16. If yes, how did you help your child overcome this fear or anxiety?
    ………………………………………….
17. Have you ever encountered a dog that showed aggressive or dominant behavior toward your child?
    a. Yes
    b. No
18. Have you ever allowed your child to approach an unfamiliar dog without first asking the owner?
    a. Yes
    b. No
19. Have your children ever expressed interest in having a dog?
    a. Yes
    b. No
20. Have you ever had a dog that displayed aggressive behavior toward children?
    a. Yes
    b. No
21. Would you allow your child to play with a dog that has not received proper training?
    a. Yes
    b. No
22. If not, why not?
    a. Safety concerns
    b. It is not worth the risk
    c. Uncertainty regarding the dog's behavior
    d. All of the above
    e. None of the above
23. Have you ever seen your child display inappropriate behavior toward a dog?
    a. Yes
    b. No
24. If yes, how did you address the situation?
    a. By punishing the child
    b. Educating the child about appropriate behavior
    c. Both A and B
    d. None of the above
25. How do you feel when your child pets an unfamiliar dog on the street?
    a. I am comfortable with this behavior
    b. I am uncomfortable with this situation
    c. It depends on the dog's behavior
    d. None of the above
26. On a scale from 1 to 10, how comfortable are you when your child interacts with unfamiliar dogs?
    a. 1 (not comfortable at all)
    b. 5 (neutral)
    c. 10 (completely comfortable)
27. Have you ever used a correction or training device to help control a dog's aggressive behavior?
    a. Yes
    b. No
28. Do you trust your ability to recognize potentially dangerous dog behavior?
    a. Yes
    b. No
29. If not, what measures would you take to ensure your child's safety?
    a. Avoiding contact with such dogs
    b. Always asking permission before petting unfamiliar dogs
    c. Educating myself about dog behavior
    d. All of the above
    e. None of the above
30. How do you think your child's experience with dogs could affect their future relationships with animals?
    a. Positively
    b. Negatively
    c. Neutral
    d. Depends on the situation
31. How do you feel about using punishment or physical force to discipline a dog?
    a. Acceptable in certain situations
    b. Never acceptable
    c. I am not sure
32. How would you react if an unfamiliar dog approached your child and appeared friendly?
    a. Allow the child to pet the dog under supervision
    b. Move the child away from the dog
    c. Ask the owner first
    d. All of the above
    e. None of the above
33. How would you react if an unfamiliar dog approached your child and appeared aggressive?
    a. Feel panic and anxiety
    b. Remain calm and immediately remove the child

c. Hide behind something or someone
d. All of the above
e. None of the above

34. What is your opinion about having pets at home?
 a. Strongly agree
 b. Mostly agree
 c. Neutral
 d. Mostly disagree
 e. Strongly disagree
35. Have you ever had a pet dog?
 a. Yes
 b. No
36. Do you believe dogs can be trained?
 a. Yes
 b. No
37. Do you believe in dogs' ability to be good companions?
 a. Absolutely
 b. For the most part
 c. Neutral
 d. Most of the time not
 e. No
38. Do you believe people should adopt dogs from shelters?
 a. Yes
 b. No
39. Have you ever adopted a dog from an animal shelter?
 a. Yes
 b. No
40. Do you currently own a dog?
 a. Yes
 b. No
41. If yes, what breed is your dog?
 ………………………………………….
42. Do you think people should own dogs exclusively for guarding purposes?
 a. Yes
 b. No
43. Have you ever been bitten by a dog?
 a. Yes
 b. No
44. How do you react when you see a stray dog?
 a. I immediately try to help
 b. I can sometimes offer help
 c. I am neutral
 d. I usually stay away from it
 e. I may intimidate it if necessary
45. Do you believe pet dog owners should be allowed to bring their animals into public places such as malls and parks?
 a. Yes
 b. No
46. Do you think certain dog breeds are more dangerous than others?
 a. Yes
 b. No
47. Do you believe small dog breeds are easier to care for than large breeds?
 a. Yes
 b. No
48. Do you think dogs should be allowed to roam freely in public places such as beaches and parks?
 a. Yes
 b. No
49. Do you think dogs should be used for scientific experiments?
 a. Yes
 b. No
50. Have you ever heard of animal-assisted therapy?
 a. Yes
 b. No
51. Do you think dog owners should train their pets to socialize with other people and animals?
 a. Yes
 b. No
52. Do you support the adoption of senior dogs?
 a. Yes
 b. No
53. Do you think dogs can be trained to detect diseases such as cancer?
 a. Yes
 b. No
54. Have you ever participated in animal-assisted therapy?
 a. Yes
 b. No
55. Do you consider animal-assisted therapy beneficial?
 a. Yes
 b. No
56. Would you consider using animal-assisted therapy as a form of treatment?
 a. Yes
 b. No
 c. I am not sure
57. Do you believe pets can help reduce stress levels?
 a. Yes
 b. No
58. Do you think animal-assisted therapy is suitable for schools?
 a. Yes
 b. No
59. If yes, which age group do you think would benefit the most from animal-assisted therapy?
 a. Elementary school students
 b. Middle school students
 c. High school students
60. Do you think animal-assisted therapy sessions could distract pupils/students from their studies?
 a. Yes
 b. No
61. In your opinion, can animal-assisted therapy improve pupils'/students' mental health?
 a. Yes
 b. No
62. What mental/emotional health problems do you think animal-assisted therapy could help with?
 ………………………………………….
63. Do you think animal-assisted therapy can help pupils/students with learning difficulties?
 a. Yes
 b. No
64. What learning difficulties do you think animal-assisted therapy could help with?
 ………………………………………….
65. Do you believe animal-assisted therapy can help create a more positive school environment?
 a. Yes
 b. No
66. Do you think allergies to animals could be a concern for students during therapy sessions?
 a. Yes
 b. No
67. If yes, what solution would you suggest for this issue?
 a. Using only hypoallergenic animals
 b. Completely avoiding animal-assisted therapy sessions
 c. Offering alternative therapy sessions
68. Do you believe animal-assisted therapy can improve the relationship between pupils/students and teachers?
 a. Yes
 b. No
69. Do you think animal-assisted therapy can help reduce absenteeism in schools?
 a. Yes
 b. No
70. Would you be interested in participating in animal-assisted therapy sessions?
 a. Yes
 b. No

71. Do you believe a specific animal-assisted therapy course is necessary for staff and volunteers participating in these sessions?
    a. Yes
    b. No
72. Do you consider animal-assisted therapy a cost-effective way to improve pupils'/students' mental health?
    a. Yes
    b. No
73. How often do you think animal-assisted therapy sessions should take place during the school year?
    a. Once a week
    b. Several times a month
    c. Several times per semester
    d. Other
74. Would you prefer to have a permanent therapy animal in the educational institution or different animals brought each time?
    a. Permanent therapy animal
    b. Different animals each time
75. Do you think parents would support animal-assisted therapy sessions in schools?
    a. Yes
    b. No
76. In your opinion, do you think some pupils/students might feel anxious around animals?
    a. Yes
    b. No
77. Do you think animal-assisted therapy can help pupils/students cope better with pressures from the educational environment?
    a. Yes
    b. No
78. Do you think pupils/students would be interested in participating in animal-assisted therapy sessions?
    a. Yes
    b. No
79. What is your biggest concern if animal-assisted therapy sessions were organized in school?
    a. Safety concerns
    b. Allergy-related concerns
    c. Difficulties related to preparing therapy sessions
    d. Other
80. If you had to describe animal-assisted therapy in a few words, what would you say?
    a. Good mood
    b. Relaxation
    c. Therapeutic
    d. Fun
    e. Other
81. Do you think animal-assisted therapy sessions should be mandatory for all pupils/students?
    a. Yes
    b. No
82. In your opinion, do you think animal-assisted therapy could also be beneficial for teachers?
    a. Yes
    b. No
