# Supplementary material for: Perceptions of dogs and animal-assisted support programs among veterinary students and teaching staff in Romania
Source: Front Vet Sci. 2026 Jul 29;13:1871706. doi: 10.3389/fvets.2026.1871706 (PMC13461325; doi:10.3389/fvets.2026.1871706)
Supplement: Supplementary file 2 [file Data_Sheet_2.pdf]

Teaching Staff Questionnaire

1. What is your gender?
   a. Male
   b. Female
2. What is your age?
   a. Under 25 years old
   b. 25–35
   c. 36–45
   d. 46–55
   e. 56–60
   f. Over 60
3. Please specify your profession: ………………
4. How long have you been a university faculty member?
   a. Less than 1 year
   b. 1–5 years
   c. 5–10 years
   d. More than 10 years
5. How would you describe your teaching style?
   a. Traditional
   b. Student-centered
   c. Lecture-based
   d. Other
6. Do any of your students have special needs?
   a. Yes, physical disabilities
   b. Yes, learning difficulties
   c. Yes, emotional/behavioral disorders
   d. Yes, other special needs: ………………….
   e. No
7. Which year of study do you teach most frequently?
   a. First year
   b. Second year
   c. Third year
   d. Fourth year
   e. Fifth year
   f. Sixth year
8. What percentage of your students are international students?
   a. Between 0–5%
   b. Between 6–10%
   c. Between 11–20%
   d. Between 21–30%
   e. More than 30%
9. What is the average age of your students?
   a. 18–20 years
   b. 21–23 years
   c. 24–26 years
   d. 27–29 years
   e. Over 30 years
10. What percentage of your students have a disability or require accommodations?
    a. Between 0–5%
    b. Between 6–10%
    c. Between 11–20%
    d. Between 21–30%
    e. More than 30%
11. What is the gender distribution in a year of study?
    a. Predominantly male
    b. Slightly more males than females
    c. Equal gender distribution
    d. Slightly more females than males
    e. Predominantly female
12. How often do you observe signs of mental health problems among your students?
    a. Always
    b. Frequently
    c. Sometimes
    d. Rarely
    e. Never
13. Do you feel prepared to respond to and support students' mental health needs?
    a. Yes, always
    b. Most of the time
    c. Somewhat, but I would need additional resources or training
    d. Not really, I am not sure how to support students with mental health problems
    e. No, not at all
14. What types of resources or training do you believe would be useful for addressing and supporting students' mental health?
    a. Access to a mental health professional on campus
    b. Professional development opportunities related to mental health support
    c. Additional training or education in mental health first aid
    d. Improved communication and collaboration with campus mental health services
    e. All of the above
15. What percentage of students have experienced mental health problems in the past year?
    a. Less than 5%
    b. Between 5–10%
    c. Between 10–20%
    d. Between 20–30%
    e. More than 30%
16. Do you have children?
    a. Yes
    b. No
17. Have you talked to your children about safe interactions with dogs?
    a. Yes
    b. No
18. Has your child ever expressed fear or anxiety regarding dogs?
    a. Yes
    b. No
19. If yes, how did you help your child overcome this fear or anxiety?
    a. I encouraged them to accept dogs in a supervised context
    b. Exposure to dogs was avoided
    c. Enrolled in therapy programs
    d. All of the above
    e. None of the above
20. Have you ever encountered a dog that showed aggressive or dominant behavior toward your child?
    a. Yes
    b. No
21. Have you ever allowed your child to approach an unfamiliar dog without first asking the dog's owner?
    a. Yes
    b. No
22. Have your children ever expressed interest in having a dog?
    a. Yes
    b. No
23. Have you ever had a dog that displayed aggressive behavior toward children?
    a. Yes
    b. No
24. Would you allow your child to play with a dog that has not received proper training?
    a. Yes
    b. No
25. If not, why not?
    a. Safety concerns
    b. It is not worth the risk
    c. Uncertainty regarding the dog's behavior
    d. All of the above
    e. None of the above
26. Have you ever seen your child display inappropriate behavior toward a dog?
    a. Yes
    b. No
27. If yes, how did you address the situation?
    a. By punishing the child
    b. Educating the child about appropriate behavior

c. Both A and B
d. None of the above

28. How do you feel when your child pets an unfamiliar dog on the street?
    a. I am comfortable with this behavior
    b. I am uncomfortable with this situation
    c. It depends on the dog's behavior
    d. None of the above
29. On a scale from 1 to 10, how comfortable are you when your child interacts with unfamiliar dogs?
    a. 1 (not comfortable at all)
    b. 5 (neutral)
    c. 10 (completely comfortable)
30. Have you ever used a correction or training device to help control a dog's aggressive behavior?
    a. Yes
    b. No
31. Do you trust your ability to recognize potentially dangerous behavior in dogs?
    a. Yes
    b. No
32. If not, what measures would you take to ensure your child's safety?
    a. Avoiding contact with such dogs
    b. Always asking permission before petting unfamiliar dogs
    c. Educating myself about dog behavior
    d. All of the above
    e. None of the above
33. How do you think your child's experience with dogs could affect their future relationships with animals?
    a. Positively
    b. Negatively
    c. Neutral
    d. Depends on the situation
34. How do you feel about using punishment or physical force to discipline a dog?
    a. Acceptable in certain situations
    b. Never acceptable
    c. I am not sure
35. How would you react if an unfamiliar dog approached your child and appeared friendly?
    a. Allow the child to pet the dog under supervision
    b. Move the child away from the dog
    c. Ask the owner first
    d. All of the above
    e. None of the above
36. How would you react if an unfamiliar dog approached your child and appeared aggressive?
    a. Feel panic and anxiety
    b. Remain calm and immediately remove the child
    c. Hide behind something or someone
    d. All of the above
    e. None of the above
37. What is your opinion about having pets at home?
    a. Strongly agree
    b. Mostly agree
    c. Neutral
    d. Mostly disagree
    e. Strongly disagree
38. Have you ever had a pet dog?
    a. Yes
    b. No
39. Do you believe dogs can be trained?
    a. Yes
    b. No
40. Do you believe in dogs' ability to be good companions?
    a. Absolutely
    b. For the most part
    c. Neutral
    d. Most of the time not
    e. No
41. Do you believe people should adopt dogs from shelters?
    a. Yes
    b. No
42. Have you ever adopted a dog from an animal shelter?
    a. Yes
    b. No
43. Do you currently own a dog?
    a. Yes
    b. No
44. If yes, what breed is your dog? ……………………….....
45. Do you think people should own dogs exclusively for guarding purposes?
    a. Yes
    b. No
46. Have you ever been bitten by a dog?
    a. Yes
    b. No
47. How do you react when you see a stray dog?
    a. I immediately try to help
    b. I can sometimes offer help
    c. I am neutral
    d. I usually stay away from it
    e. I may intimidate it if necessary
48. Do you believe pet dog owners should be allowed to bring their animals into public places such as malls and parks?
    a. Yes
    b. No
49. Do you think certain dog breeds are more dangerous than others?
    a. Yes
    b. No
50. Do you believe small dog breeds are easier to care for than large breeds?
    a. Yes
    b. No
51. Do you think dogs should be allowed to roam freely in public places such as beaches and parks?
    a. Yes
    b. No
52. Do you think dogs should be used for scientific experiments?
    a. Yes
    b. No
53. Do you think dog owners should train their pets to socialize with other people and animals?
    a. Yes
    b. No
54. Do you support the adoption of senior dogs?
    a. Yes
    b. No
55. Do you think dogs can be trained to detect diseases such as cancer?
    a. Yes
    b. No
56. Have you ever heard of animal-assisted therapy?
    a. Yes
    b. No
57. Have you ever participated in animal-assisted therapy?
    a. Yes
    b. No
58. Do you consider animal-assisted therapy beneficial?
    a. Yes
    b. No
59. Would you consider using animal-assisted therapy as a form of treatment?
    a. Yes
    b. No
60. Do you believe pets can help reduce stress levels?
    a. Yes
    b. No
    c. I am not sure
61. Do you think animal-assisted therapy is suitable for schools?
    a. Yes

    b. No

62. If yes, which age group do you think would benefit the most from animal-assisted therapy?
    a. Elementary school students
    b. Middle school students
    c. High school students
    d. Other: ....
63. Do you think animal-assisted therapy sessions could distract pupils/students from their studies?
    a. Yes
    b. No
64. In your opinion, can animal-assisted therapy improve the mental health of pupils/students?
    a. Yes
    b. No
65. What mental/emotional health problems do you think animal-assisted therapy could help with?
    ………………………………………….
66. Do you think animal-assisted therapy can help pupils/students with learning difficulties?
    a. Yes
    b. No
67. What learning difficulties do you think animal-assisted therapy could help with?
    ………………………………………….
68. Do you believe animal-assisted therapy can help create a more positive school environment?
    a. Yes
    b. No
69. Do you think animal allergies could be a significant concern for pupils/students during animal-assisted therapy sessions?
    a. Yes
    b. No
70. If yes, what solution would you suggest for this issue?
    a. Using only hypoallergenic animals
    b. Completely avoiding animal-assisted therapy sessions
    c. Offering alternative therapy sessions
71. Do you believe animal-assisted therapy can improve the relationship between pupils/students and teachers?
    a. Yes
    b. No
72. Do you think animal-assisted therapy can help reduce absenteeism in schools?
    a. Yes
    b. No
73. Would you be interested in participating in animal-assisted therapy sessions with your pupils/students?
    a. Yes
    b. No
74. Do you believe a specific animal-assisted therapy course is necessary for staff and volunteers participating in these sessions?
    a. Yes
    b. No
75. Do you consider animal-assisted therapy a cost-effective way to improve pupils'/students' mental health?
    a. Yes
    b. No
76. How often do you think animal-assisted therapy sessions should take place during the school year?
    a. Once a week
    b. Several times a month
    c. Several times per semester
    d. Other: …………………………………
77. Would you prefer to have a permanent therapy animal in the educational institution or different animals brought each time?
    a. Permanent therapy animal
    b. Different animals each time
78. Do you think parents would support animal-assisted therapy sessions in schools?
    a. Yes
    b. No
79. In your opinion, do you think some pupils/students might feel anxious around animals?
    a. Yes
    b. No
80. Do you think animal-assisted therapy can help pupils/students cope better with pressures from the educational environment?
    a. Yes
    b. No
81. Have you ever talked with your pupils/students about animal-assisted therapy?
    a. Yes
    b. No
82. If you have talked with pupils/students about animal-assisted therapy, what was their response?
    a. Positive
    b. Negative
    c. Neutral
83. Do you think pupils/students would be interested in participating in animal-assisted therapy sessions?
    a. Yes
    b. No
84. What is your biggest concern if animal-assisted therapy sessions were organized in your school?
    a. Safety concerns
    b. Allergy-related concerns
    c. Difficulties related to preparing therapy sessions
    d. Other: ....
85. If you had to describe animal-assisted therapy in a few words, what would you say?
    a. Good mood
    b. Relaxation
    c. Therapeutic
    d. Fun
    e. Other: …………………………………..
86. Do you think animal-assisted therapy sessions should be mandatory for all pupils/students?
    a. Yes
    b. No
87. In your opinion, do you think animal-assisted therapy could also be beneficial for teachers?
    a. Yes
    b. No
