# Supplementary material for: Perceptions of dogs and animal-assisted support programs among veterinary students and teaching staff in Romania
Source: Front Vet Sci. 2026 Jul 29;13:1871706. doi: 10.3389/fvets.2026.1871706 (PMC13461325; doi:10.3389/fvets.2026.1871706)
Supplement: Supplementary file 3 [file Data_Sheet_3.pdf]

Students Questionnaire

1. What is your gender?

- a. Female
- b. Male

2. What is your age?

- a. 18–21
- b. 22–25
- c. 26–30
- d. 31–35
- e. 36+

3. Are you currently enrolled in veterinary medicine faculty?

- a. Yes
- b. No, but I plan to return to school
- c. No, and I do not plan to return to school

4. How often do you exercise per week?

- a. Never
- b. Once a week
- c. 2–3 times a week
- d. 4–5 times a week
- e. More than 5 times a week

5. Have you ever been diagnosed with a mental or emotional health problem?

- a. Yes
- b. No

6. Are you currently taking prescription medication for any mental or emotional health problem?

- a. Yes
- b. No

7. What is your opinion regarding keeping pets at home?

- a. Strongly agree
- b. Mostly agree
- c. Neutral
- d. Mostly disagree
- e. Strongly disagree

8. Have you ever had a pet dog?

- a. Yes
- b. No

9. Do you consider dogs good companion animals?

- a. Strongly agree
- b. Agree
- c. Neutral
- d. Disagree
- e. Strongly disagree

10. Do you believe people should adopt dogs from shelters?

- a. Yes
- b. No

11. Have you ever adopted a dog from an animal shelter?

- a. Yes
- b. No

12. Do you currently own a dog?

- a. Yes
- b. No

13. Have you ever been bitten by a dog?

- a. Yes
- b. No

14. Do you believe pet dog owners should be allowed to bring their animals into public places such as malls and parks?

- a. Yes
- b. No

15. Do you believe certain dog breeds may present a higher risk of aggressive behavior than others?

- a. Yes
- b. No

16. Do you believe small dog breeds are easier to care for than large breeds?

- a. Yes
- b. No

17. Do you think dogs should be allowed to roam freely in public places such as beaches and parks?

- a. Yes
- b. No

18. Do you believe dogs should be used in scientific research?

- a. Yes
- b. No

19. Do you support the adoption of senior dogs?

- a. Yes
- b. No

20. Do you think dog owners should train their pets to socialize with other people and animals?

- a. Yes
- b. No

21. Have you ever heard of Animal-Assisted Support Programs (AASP)?
- a. Yes
- b. No

22. Have you ever participated in Animal-Assisted Support Programs (AASP)?
- a. Yes
- b. No

23. Do you consider Animal-Assisted Support Programs (AASP) beneficial?
- a. Yes
- b. No

24. Do you consider that Animal-Assisted Support Programs (AASP) can be a treatment option?
- a. Yes
- b. No

25. Do you believe pets can help reduce stress levels?
- Yes
- No

26. Do you believe Animal-Assisted Support Programs (AASP) are suitable for educational institutions?
- a. Yes
- b. No

27. If yes, which age group do you think would benefit the most from animal- assisted therapy?
- a. Primary education
- b. Lower and upper secondary education
- c. Higher education
- d. I don’t really know

28. Would you be interested in participating in AASP?
- a. Yes
- b. No

29. In your opinion, AASP could also be beneficial for teachers?
- a. Yes
- b. No

30. Do you think AASP sessions could distract students from their studies?
- a. Yes
- b. No

31. In your opinion , can AASP improve students’ mental health?
- a. Yes
- b. No

32. Do you think AASP can help students with learning difficulties?
- a. Yes
- b. No

33. Do you believe AASP can help create a more positive school environment?
- a. Yes
- b. No

34. Do you think AASP can help reduce dropout rates?
- a. Yes
- b. No

35. Do you believe a specific AASP course is necessary for staff and volunteers participating in these sessions?
- a. Yes
- b. No

36. Do you consider AASP a cost- effective way to improve students’ mental health?
- a. Yes
- b. No

37. How often do you think AASP sessions should take place during the academic year?

a. Once a week or several times a month

b. Several times per semester

c. Once per semester or year

d. I have no opinion

38. Would you prefer to have a permanent support animal in the educational institution or different animals brought each time?
- a. Permanent animal
- b. Different animals each time

39. In your opinion, do you think some students might feel anxious around animals?
- a. Yes
- b. No

40. Do you think AASP can help students cope better with pressures from the educational environment?
- a. Yes
- b. No

41. Do you think AASP sessions should be mandatory for all students?
- a. Yes
- b. No
