# Supplementary material for: Perceptions of dogs and animal-assisted support programs among veterinary students and teaching staff in Romania
Source: Front Vet Sci. 2026 Jul 29;13:1871706. doi: 10.3389/fvets.2026.1871706 (PMC13461325; doi:10.3389/fvets.2026.1871706)
Supplement: Supplementary file 4 [file Data_Sheet_4.pdf]

Teaching Staff Questionnaire

1. What is your gender?
a. Male
b. Female
2. What is your age?
a. Under 25 years old
b. 25–35
c. 36–45
d. 46–55
e. 56–60
f. Over 60
3. How long have you been a university faculty member?
a. Less than 1 year
b. 1–5 years
c. 5–10 years
d. More than 10 years
4. How would you describe your teaching style?
a. Traditional
b. Student-centered
c. Lecture-based
d. Other
5. Do any of your students have special needs?
a. Yes, physical disabilities
b. Yes, learning difficulties
c. Yes, emotional/behavioral disorders
d. Yes, other special needs: ………………….
e. No
6. What percentage of your students have a special need or require accommodations?
a. Between 0–5%
b. Between 6–10%
c. Between 11–20%
d. Between 21–30%
e. More than 30%
7. How often do you observe signs of mental or emotional health problems among your students?
a. Always
b. Frequently
c. Sometimes
d. Rarely
e. Never
8. Do you feel prepared to respond and to support students' mental health needs?
a. Yes, always
b. Most of the time
c. Somewhat, but I would need additional resources or training
d. Not really, I am not sure how to support students with mental health problems
e. No, not at all
9. What types of resources or training do you believe would be useful for addressing and supporting students' mental health?
a. Access to a mental health professional on campus
b. Professional development opportunities related to mental health support
c. Additional training or education in mental health first aid
d. Improved communication and collaboration with campus mental health services
e. All of the above
10. What percentage of students have you noticed to experience mental health problems, in the past year?
a. Less than 5%
b. Between 5–10%
c. Between 10–20%
d. Between 20–30%
e. More than 30%
11. What is your opinion regarding keeping pets at home?
a. Strongly agree
b. Mostly agree
c. Neutral
d. Mostly disagree
e. Strongly disagree
12. Have you ever had a pet dog?
a. Yes
b. No
13. Do you consider dogs good companion animals?
a. Strongly agree
b. Agree
c. Neutral
d. Disagree
e. Strongly disagree
14. Do you believe people should adopt dogs from shelters?
a. Yes
b. No
15. Have you ever adopted a dog from an animal shelter?
a. Yes
b. No
16. Do you currently own a dog?
a. Yes
b. No
17. Have you ever been bitten by a dog?
a. Yes
b. No
18. Do you believe pet dog owners should be allowed to bring their animals into public places such as malls and parks?
a. Yes
b. No
19. Do you believe certain dog breeds may present a higher risk of aggressive behavior than others?
a. Yes
b. No
20. Do you believe small dog breeds are easier to care for than large breeds?
a. Yes
b. No
21. Do you think dogs should be allowed to roam freely in public places such as beaches and parks?
a. Yes
b. No
22. Do you believe dogs should be used in scientific research?
a. Yes
b. No
23. Do you think dog owners should train their pets to socialize with other people and animals?
a. Yes
b. No
24. Do you support the adoption of senior dogs?
a. Yes
b. No
25. Have you ever heard of Animal-Assisted Support Programs (AASP)?
a. Yes
b. No
26. Have you ever participated in Animal-Assisted Support Programs (AASP)?
a. Yes
b. No

27. Do you consider Animal-Assisted Support Programs (AASP) beneficial?
a. Yes
b. No
28. Do you consider that Animal-Assisted Support Programs (AASP) can be a treatment option?
a. Yes
b. No
29. Do you believe pets can help reduce stress levels?
a. Yes
b. No
30. Do you believe Animal-Assisted Support Programs (AASP) are suitable for educational institutions?
a. Yes
b. No
31. If yes, which age group do you think would benefit the most from AASP?
a. Primary education
b. Lower and upper secondary education
c. Higher education
d. I don't really know
32. Do you think students would be interested in participating in AASP?
a. Yes
b. No
33. Would you be interested in participating in AASP with your students?
a. Yes
b. No
34. Do you think AASP could distract students from their studies?
a. Yes
b. No
35. In your opinion, can AASP improve the mental health of students?
a. Yes
b. No
36. Do you think AASP can help students with learning difficulties?
a. Yes
b. No
37. Do you believe AASP can help create a more positive school environment?
a. Yes
b. No
38. Do you think AASP can help reduce dropout rates?
a. Yes
b. No
39. Do you believe a specific AASP course is necessary for staff and volunteers participating in these sessions?
a. Yes
b. No
40. Do you consider AASP a cost-effective way to improve students' mental health?
a. Yes
b. No
41. How often do you think AASP sessions should take place during the academic year?
a. Once a week or several times a month
b. Several times per semester
c. Once per semester or year
d. I have no opinion
42. Would you prefer to have a permanent support animal in the educational institution or different animals brought each time?
a. Permanent animal
b. Different animals each time
43. In your opinion, do you think some students might feel anxious around animals?
a. Yes
b. No
44. Do you think AASP can help students cope better with pressures from the educational environment?
a. Yes
b. No
45. Do you think AASP sessions should be mandatory for all students?
a. Yes
b. No
